# Balancing the skin: antagonistic ubiquitin-like signaling pathways govern epidermal homeostasis

**DOI:** 10.1038/s41392-026-02960-6

**Published:** 2026-09-30

**Authors:** Katrin Rietscher, Benjamin Klein

**Affiliations:** 1 Department of Dermatology, Allergology and Venereology, Leipzig University Medical Center, University of Leipzig, Leipzig, Germany; 2 Division of Rheumatology, Department of Internal Medicine, University of Michigan, Ann Arbor, MI USA

**Keywords:** Cell biology, Molecular biology

**Fig. 1:**
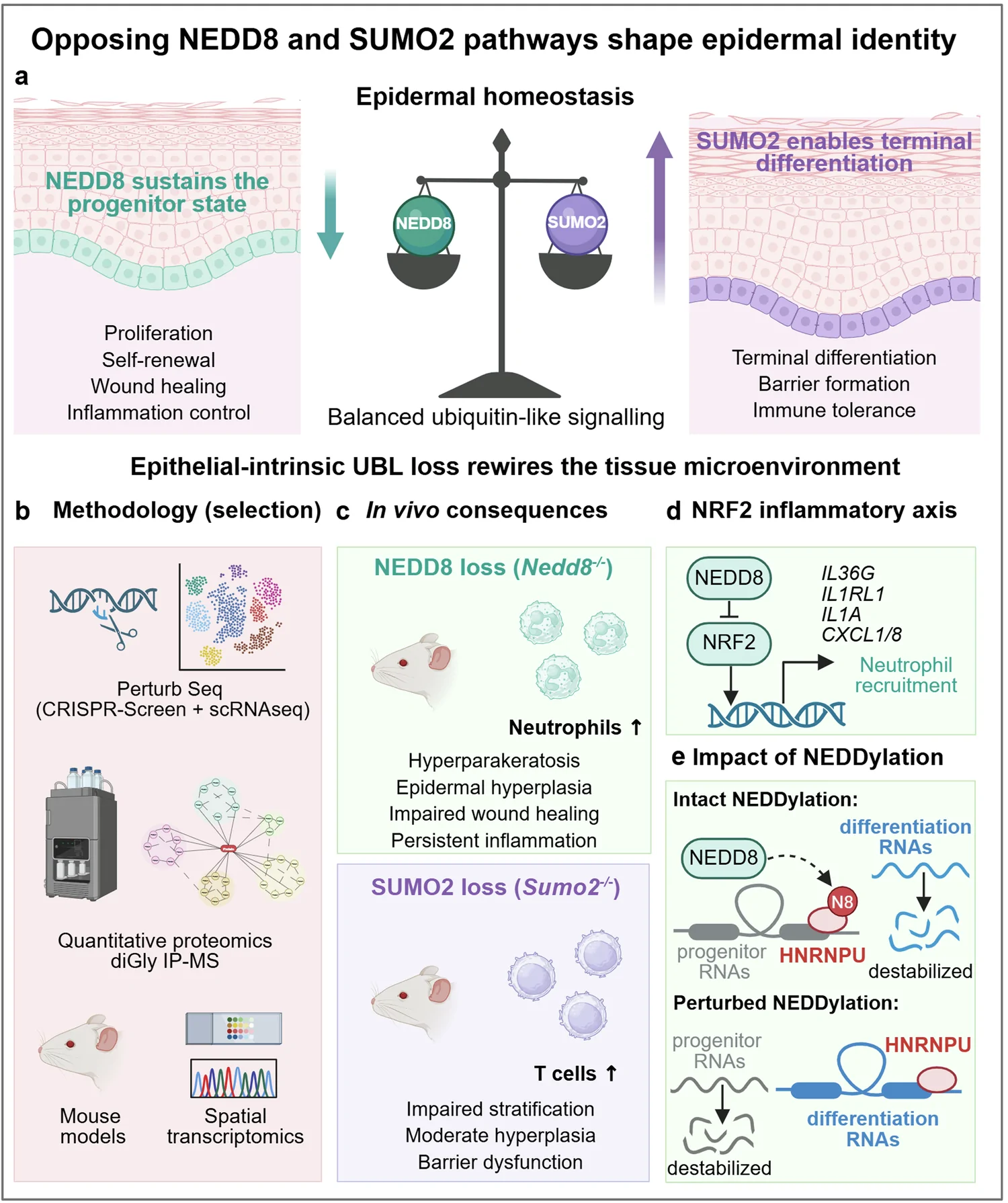
Antagonistic NEDD8 and SUMO2 signaling regulates epidermal homeostasis, regeneration, and inflammatory responses. **a** Balanced ubiquitin-like (UBL) signaling maintains epidermal homeostasis by coordinating the opposing functions of NEDD8 (Neural precursor cell expressed developmentally downregulated protein 8) and SUMO2 (Small ubiquitin-like modifier 2). NEDD8 preserves the progenitor state, supporting proliferation, self-renewal, wound healing, and inflammatory control, whereas SUMO2 promotes terminal keratinocyte differentiation, epidermal barrier formation, and immune tolerance. **b** Selection of the multiomic and functional approaches used in this study, including quantitative proteomics, di-glycine remnant immunoprecipitation-mass spectrometry (diGly IP-MS), Perturb-seq, spatial transcriptomics, and in vivo genetic models. **c** Keratinocyte-specific deletion of *Nedd8* induces premature differentiation, epidermal hyperplasia, impaired wound healing, persistent inflammation, and neutrophil recruitment, whereas *Sumo2* deficiency disrupts epidermal stratification, compromises barrier function, and promotes dermal T-cell infiltration. **d** Mechanistically, NEDD8 loss activates NRF2-dependent inflammatory signaling, inducing expression of neutrophil-recruiting mediators, including *IL36G*, *IL1RL1*, *IL1A*, and *CXCL1/8*. **e** Beyond its canonical role in ubiquitin-proteasome signaling, NEDDylation regulates HNRNPU (Heterogeneous nuclear ribonucleoprotein U)-dependent RNA stabilization. Intact NEDDylation promotes stabilization of progenitor-associated transcripts, whereas loss of NEDDylation redirects HNRNPU toward differentiation-associated mRNAs, thereby controlling epithelial cell fate. Figure created with BioRender.com

In a recent study published in *Science*, Winge et al. identified the ubiquitin-like modifiers NEDD8 and SUMO2 as antagonistic regulators of epidermal homeostasis, revealing an unexpected role for ubiquitin-like signaling in epidermal cell fate and tissue repair (Fig. 1a).^1^

The epidermis forms a highly specialized barrier that protects the body from mechanical stress, pathogens, and environmental insults. Epidermal homeostasis is maintained through continuous renewal of the stratified epithelium, in which basal progenitor cells self-renew while generating keratinocytes that migrate through the suprabasal layers to replenish the epidermal barrier.^2,3^ This process relies on coordinated molecular signaling, mechanical forces, and adhesive remodeling that drive keratinocyte differentiation, tissue stratification, and renewal.^4^ Upon tissue damage or infection, immune surveillance and inflammatory signaling rapidly restore tissue integrity. Disruption of these processes compromises barrier integrity and contributes to chronic inflammation, impaired wound healing, and tumorigenesis.

Although transcriptomic and epigenetic studies have provided important insights into the regulatory networks affecting keratinocyte differentiation, proteomic diversity across epidermal cell states suggests posttranslational modifications (PTMs) as another regulator of tissue states. This prompted Winge and colleagues to investigate differentiation-associated proteome remodeling. By integrating Perturb-seq, quantitative proteomics, di-glycine remnant immunoprecipitation-mass spectrometry (diGly IP-MS), spatial transcriptomics, and in vivo genetic models, Winge et al. established a comprehensive multiomic framework to map posttranslational regulatory networks during keratinocyte differentiation (Fig. 1b).

DiGly IP-MS enriches peptides carrying a characteristic di-glycine remnant left on lysine residues after tryptic digestion of ubiquitin- or ubiquitin-like protein (UBL)-modified substrates, enabling proteome-wide identification of UBL-modified peptides. Using this approach, the authors found that proteins progressively lost during in vitro differentiation of primary human keratinocytes were enriched for diGly modifications. By contrast, the core ubiquitination machinery remained unchanged, suggesting that selective ubiquitin-like signaling pathways regulate proteome remodeling during epithelial differentiation.

To define the role of ubiquitin-related networks in differentiated keratinocytes, Winge et al. performed a Perturb-seq screen, combining CRISPR-based gene perturbation with single-cell RNA sequencing. This screen identified NEDDylation and SUMOylation as two ubiquitin-like pathways with opposing roles in epidermal differentiation: perturbation of NEDDylation components induced differentiation, whereas disruption of the SUMOylation machinery prevented it.

NEDDylation is a PTM that conjugates the ubiquitin-like protein NEDD8 (Neural precursor cell expressed developmentally downregulated protein 8) to target proteins. Classically, NEDD8 has been viewed as an activator of cullin-RING ubiquitin ligases, where modification of cullin scaffold proteins stimulates ubiquitination and subsequent proteasomal degradation of regulatory proteins.^5^ By contrast, SUMOylation conjugates SUMO (Small ubiquitin-like modifier) to lysine residues of target proteins, primarily regulating protein activity, subcellular localization, protein-protein interactions, and transcriptional responses, thereby influencing diverse cellular processes, including differentiation and stress adaptation. The opposing functions of NEDD8 and SUMO2 were confirmed in primary human keratinocytes and 3D skin organoids using complementary genetic and pharmacological approaches.

The relevance of these findings in vivo was confirmed in tamoxifen-inducible keratinocyte-specific knockout mice. Loss of *Nedd8* caused striking epidermal abnormalities, including hyperkeratosis, epidermal hyperplasia, and premature differentiation, consistent with accelerated loss of progenitor identity. By contrast, *Sumo2* deletion impaired epidermal stratification and reduced expression of differentiation markers, indicating defective terminal differentiation. Despite these opposite effects on epidermal differentiation, both knockout models showed impaired barrier function, reflected by increased transepidermal water loss, highlighting the role of epidermal homeostasis in skin barrier integrity. Notably, *Nedd8* loss also impaired wound healing, resulting in defective re-epithelialization, persistent expression of immature barrier transcripts at the wound edge, and increased neutrophil infiltration, pointing to an additional role in coordinating regenerative and inflammatory responses (Fig. 1c).

As disruption of epidermal homeostasis likely affects the cutaneous microenvironment, the authors performed single-cell and spatial transcriptomic analyses to reveal distinct immune landscapes in the two knockout models. *Nedd8*-deficient murine skin showed marked neutrophil accumulation, whereas *Sumo2* loss led to dermal T cell infiltration. These immune changes were accompanied by stromal remodeling involving transcriptional changes in fibroblasts and macrophages, highlighting extensive epithelial-stromal crosstalk. Together, these findings suggest that changes in epithelial cell identity not only occur in response to inflammation but actively shape immune recruitment and tissue organization. This study reinforces the emerging concept that epithelial cell-state transitions are key determinants of microenvironmental composition, with intrinsic dysregulation of keratinocyte pathways central to innate immune response regulation (Fig. 1c).

As *Nedd8*-deficient mice exhibited impaired wound healing, the authors performed mechanistic analyses to identify the underlying pathways. Confocal microscopy, spatial transcriptomics, and genome-wide NRF2 CUT&RUN profiling in primary human keratinocytes revealed that *Nedd8* loss triggered aberrant activation of NRF2, a key regulator of oxidative stress responses, resulting in increased expression of pro-inflammatory mediators linked to neutrophil recruitment. Genetic ablation of *Nrf2* attenuated inflammatory features without rescuing the differentiation defect, demonstrating that NEDD8 controls epidermal homeostasis through separable NRF2-dependent inflammatory and NRF2-independent differentiation programs (Fig. 1d).

Further, the authors found broad UPS-independent functions of NEDD8, as most proteomic changes could not be explained by conventional UPS perturbation. Surprisingly, NEDD8-dependent proteome remodeling strongly correlated with changes in RNA abundance and transcript stability, suggesting an additional role in posttranscriptional regulation. A genome-wide suppressor CRISPR screen identified the RNA-binding protein HNRNPU (Heterogeneous nuclear ribonucleoprotein U) as a central mediator of this pathway. Mechanistically, NEDDylation altered the RNA-binding repertoire of HNRNPU, promoting stabilization of progenitor- and cell cycle-associated transcripts. In contrast, loss of NEDDylation shifted HNRNPU toward binding and stabilizing pro-differentiation mRNAs (Fig. 1e).

Although the molecular consequences of NEDD8 loss are characterized in remarkable detail, several important questions remain. The mechanistic basis of SUMO2 signaling remains to be defined beyond the observation that the transcription factor TEAD1 may act as a potential SUMOylation-dependent effector. Moreover, the downstream pathways linking SUMO2 to terminal differentiation and immune regulation remain to be elucidated. Another important question is whether NEDD8 and SUMO2 are dysregulated in human inflammatory skin diseases with impaired epidermal homeostasis, such as psoriasis or atopic dermatitis.

Taken together, this study highlights ubiquitin-like signaling as a new regulator of epithelial homeostasis. Winge et al. show that NEDD8 and SUMO2 balance progenitor maintenance and differentiation, while influencing regeneration and inflammation. Given that pharmacological modulators of these pathways already exist, it will be interesting to explore whether targeting NEDDylation or SUMOylation could improve skin repair or treat inflammatory skin diseases.
